# Vulnerability to health and well-being of internally displaced persons (IDPs) in Myanmar post-military coup and COVID-19

**DOI:** 10.1186/s13690-023-01204-1

**Published:** 2023-10-21

**Authors:** Tual Sawn Khai

**Affiliations:** 1 School of Graduate Studies, Lingnan University, Hong Kong (SAR), People’s Republic of China; 2 Refugee Law Initiative (RLI), School of Advanced Study, University of London, London, UK

**Keywords:** COVID-19, Myanmar, Military coup, Internally displaced persons (IDPs), Armed conflict, Humanitarian Crisis

## Abstract

Across the globe, the COVID-19 pandemic has aggravated particular challenges for internally displaced people (IDPs). Over 1.9 million people in Myanmar have been displaced due to the escalation of armed conflict after the military coup in 2021. The vulnerability faced by IDPs in Myanmar, coupled with the impact of the recent military coup and the ongoing COVID-19 pandemic, has received little global attention. This study examined how military coup exacerbated the health and well-being of IDPs in Myanmar post the military coup. The study employed purposive sampling and Non-Government Organisations (NGOs) referrals to find participants. Qualitative in-depth telephone interviews were conducted with a total of 17 IDPs. A thematic analysis of the findings indicates that IDPs experience anxiety and fear daily, adversely affecting their mental health due to the increased escalation of armed conflict and attacks on civilian places, including IDPs shelters. Some IDPs contract COVID-19 and suffer from malaria and dengue fever owing to their precarious living conditions. Moreover, the military’s restrictions on humanitarian aid distribution, including healthcare, medicine, and food, have severely impacted the health and well-being of IDPs in Myanmar, exacerbating food shortages and limiting healthcare access.

**Supplementary Information:**

The online version contains supplementary material available at 10.1186/s13690-023-01204-1.

**Table:** 

**Text box 1. Contributions to the literature**
• This study offers unique insights into the health and well-being of IDPs in the Myanmar post-military coup, which differs from other IDP studies focusing on different regions or causes of displacement.
• This study highlights that IDPs in Myanmar are constantly living in terror from military attacks, including sexual violence used as a weapon of war, which negatively affects their mental health.
• IDPs in Myanmar urgently need humanitarian support since the coup has exacerbated their vulnerability to food insecurity and increased the risk of contracting infectious diseases and poor health conditions.

## Introduction

According to the United Nations High Commissioner for Refugees, more than 82.4 million global population have been forcibly displaced because of armed conflict, human rights violations, and environmental catastrophes [1]. In 2020, 55 million of the world’s population were internally displaced persons (IDPs), of whom 48 million were displaced due to conflict and violence, and 7 million were displaced from natural disasters [2]. The term internally displaced people (IDP) refers to “individuals or groups of individuals who have been displaced from their homes or places of habitual residence because of armed conflict, human rights violations, or natural or human-made disasters, and have not crossed an internationally recognised state border’ [3, 4].

Myanmar has engaged in one of the world’s longest civil wars since its independence in 1948. Political unrest and armed conflict between military and ethnic groups forced hundreds of civilians to flee their homes. At the end of December 2020, approximately 505,000 of the country’s population had IDP status [5]. Moreover, the ongoing armed conflict between military and civilian anti-coup forces, ethnic armed groups, and natural disasters has displaced more than 1.9 million people in Myanmar after the military coup in February 2021 [6]. Despite a lack of reliable statistics, over half of the population comprises children, older persons, and women, including pregnant women [7]. An estimated 80 per cent of the country’s 330 townships have been affected by armed conflict, with over 2,940 civilians killed and over 17,572 arrested or detained by military force, according to the United Nations reported in March 2023 [8]. However, neighbouring countries, such as the Thai and Indian governments, refused to accept those who had entered to seek protection and were further deported. For example, Thai authorities have refused to accept over 3,000 Karen ethnic people who crossed the Salween River for refuge on the border of Thailand after the Myanmar military attacked civilians with heavy weapons and aerial bombardment [9]. Similarly, the Indian government refused to accept over 20,000 Chin people who fled to Mizoram State [10]. Instead, it deployed border security forces to block Myanmar from entering India [11].

Studies have indicated that IDPs are globally more vulnerable than refugees in terms of psychological well-being, access to essentials, and protection due to reliance on government authorities, which may result in displacement [12, 13]. They will likely experience trauma, post-traumatic stress disorder, anxiety, depression, psychosocial distress, and stigma [14]. In principle, IDPs have the right to access social protection but often experience significant obstacles in accessing it in practice [15]. They face structural and institutional barriers to accessing social protection and essential services [16, 17]. For example, healthcare services are limited in displacement areas owing to a lack of healthcare facilities, healthcare professionals, and medical supplies [18, 19], geographic challenges, lack of designated healthcare centres, and discrimination in host communities [20].

Consequently, they are vulnerable to infectious diseases, malnutrition, food insecurity, limited access to reproductive healthcare, and chronic health conditions [14, 20]. Malaria, dengue fever, diarrhoea, and malnutrition are particularly prevalent among children [21–23]. In particular, the COVID-19 pandemic has exacerbated the vulnerability of IDPs and placed them at greater risk of contracting the disease due to their precarious living conditions, insufficient hygiene, and inadequate healthcare services [24–26].

Meanwhile, the number of internally displaced persons in Myanmar increased significantly since the military coup in February 2021. A recent study on developing and implementing a psychosocial support program for internally displaced persons (IDPs) in Myanmar during the COVID-19 pandemic indicated that community-based psychosocial support programs are crucial for IDPs during crises [27]. The United Nations reported that precarious living conditions urgently require critical and lifesaving assistance [6]. However, no imperial studies have been conducted on the plights of IDPs and their access to health care, particularly since the COVID-19 pandemic.

## Research objectives

This study explored how the coup d’état in Myanmar exacerbated the vulnerability and barriers to accessing healthcare among IDPs in Myanmar. The findings of this study provide practical policy implications for national authorities, international communities, and organisations to address the challenges faced by IDPs in Myanmar, including the provision of humanitarian aid to meet the basic needs of shelter, food, vaccines, and healthcare services to prevent infectious diseases among IDPs and promote their overall well-being.

### Research questions

1. What are the primary challenges faced by IDPs in Myanmar?
2. How do these impact their mental health and well-being?

## Methodology

### Study design and participants

This study was based on qualitative, online, and in-depth telephonic interviews. Interviews were conducted between June and September 2022. Owing to the sensitive nature of the topic and the hard-to-reach populations, the researcher recruited participants through referrals with the help of several non-governmental organisations (NGOs) and snowball sampling. The researcher contacted several local NGOs and international organisations to look for potential participants as part of the sampling process. Several IDP camps were approached through the IDPs’ Facebook pages or contact addresses, along with the study aims, objectives, and cover letter attachments. Neither formal nor informal relationships existed before the researcher contacted the participants.

After obtaining consent and contact information from potential participants, the researcher contacted the IDP camp leaders via phone calls, WhatsApp, or Signal, all providing end-to-end encryption. As part of the interview, the researcher explained the study’s objectives and withdrawal procedures to the participants at any stage without hesitation. Additionally, after interviewing the camp leaders, the researcher relied on them to recruit participants from the IDPs in their camps. Participants had to be at least 18 years old and reside in an IDP camp to be eligible for participation. The researcher interviewed only the IDPs who consented to participate voluntarily. An average of 20–35 min were spent on each interview. A researcher conducted all interviews in Myanmar and recorded them in audio format. The researcher made notes during interviews. As suggested by the guidelines for conducting interviews, discussions were concluded when the narratives were repeated or the data were saturated [28].

### Data collection and analysis

The researcher conducted all interviews in the Myanmar language. Following the guidelines proposed by Braun and Clarke [29, 30], the researcher derived themes through a reflective thematic analysis through an active process of interpretation and analysis. The themes were developed, created, or constructed based on carefully examining the data and the researcher’s reflective engagement with the data. To familiarise themselves with the data, the researcher thoroughly engaged with the audio recording by listening multiple times and reading the notes before transcribing them. Afterwards, the interview was transcribed verbatim into English and checked against the audio recording for authenticity. Moreover, to enhance the credibility and validity of the data, the researcher consulted an international English teacher in Thailand who was bilingual in both Myanmar and English (requested to keep anonymous *** for personal safety). After data validation and confirmation, the researcher read the transcribed data word by word and started coding by assigning initial codes to the data segments that captured meaningful information. The researcher then analysed the coded data thematically by merging similarities into a unified theme.

### Research ethical consideration

Before conducting the study, ethical approval was obtained from the Postgraduate Student Committee (PSC) of the Research Ethics Committee of Lingnan University in Hong Kong. In line with ethical research guidelines, oral consent was obtained from the participants before conducting the interviews, and they were informed that their identity and location would remain confidential throughout the research process. The IDP camp’s names, ages, and locations were omitted to protect confidentiality and keep participants’ identities anonymous. Pseudonyms were assigned to participants throughout the transcription, coding, and data analysis stages.

## Results

### Participants demographic characteristics

The study involved the participation of seven camp leaders and ten internally displaced persons (IDPs) from the Chin, Karen, and Kachin states in Myanmar. All camp leaders were male, seven of the IDPs were female, and three were male. To ensure anonymity in direct quotations, the researcher used the identifiers “IDP L1, L2” for IDP camp leaders and “IDP F1, F2” or “IDP M1, M2” for female and male IDP participants, respectively, based on their gender identification.

### Experiencing constant fears and anxieties

Since the military coup, armed conflict between ethnic groups and Myanmar’s military has escalated significantly. The military randomly raided several homes and interrogated innocent civilians, particularly at night. Several civilians were killed during the interrogation, and some of their bodies never returned to their families. Military forces use sexual violence against civilian populations as weapons. This poses a specific risk to the psychological health of women and children.

“Since the military has taken control of the state, we are in greater danger and are less secure wherever we go. The military forces have raped women and tortured them to death in many places”- IDP F1.

“At night, we are more afraid of what will happen to us. This is especially stressful if the dog is barked. No one can sleep or know what to do “- IDP F2.

Furthermore, the Myanmar military carried out indiscriminate attacks on unarmed civilians, including the IDP camps. They fear heavy weapons attacks because armed conflicts frequently occur near shelters. Because of this adversity, the mental health and well-being of the IDP population are negatively affected.

“During my wife’s child delivery in the IDP camp, I heard a military helicopter in the sky. I was terrified because I could not relocate my wife immediately to the bunker. I dug to protect myself from military airstrikes. Furthermore, the bunker was not constructed systematically, and as a result, cannot provide us with protection from an explosion if the military attacks us”- IDP M7.

“When we hear the sound of a military jet, we rush into the bunker, much like chickens fleeing into bushes when they see an eagle bird hovering above. Some children were discontent with this situation and cried inside the bunker. My feelings of upset and terror cannot be adequately expressed in the words “**-** IDP M3.

“Our lives were filled with uncertainty daily. Older adults and children are particularly vulnerable and insecure. There is never a state of calm or peace in my mind. I think about where we should repeatedly flee to safeguard our lives. We live every moment with terror and vigilance in our minds”- IDP F5.

This indicates that the military actions have significantly worsened the precarious living conditions and insecurity among IDPs in Myanmar and instilled a constant fear for their safety. Moreover, participants have expressed deep concerns regarding the heightened vulnerability of children and women in the aftermath of the military coup. The pervasive fear of being targeted and attacked persists among them. Tragically, the history of sexual abuse perpetrated by the military, such as the cases of rape and killings of women in different regions, including the Rohingya women in Rakhine State in 2017, further exacerbates their Challenges.

### Precarious living and infectious disease

Many displaced persons were newly displaced, living in precarious conditions in the jungle because of armed conflicts after the military coup. A Chin State camp leader overseeing approximately 140 IDP houses reported that hazardous living conditions and unsanitary drinking water caused many health problems.

“Currently, we are experiencing many difficulties. Our camp is constructed from anything we can get in the jungle, such as banana leaves. When it rains, our tent does not protect us from downpours. Furthermore, forest steam was used as the drinking water. The water became dirty and muddy after the rain. However, we were unable to obtain clean drinking water” IDP L1.

Participants from different locations report similar circumstances and hygiene problems.

“Many of our people suffer from stomach problems and numerous illnesses due to the lack of a hygienic toilet along with unhygienic drinking conditions in the camps”- IDP L3.

Furthermore, near the Thai border, another IDP camp leader from Karen State stated that unsanitary water and unprotected shelters put people at a higher risk of contracting various diseases.

“Our location in the jungle is a breeding ground for disease transmission, particularly malaria and dengue fever, caused by mosquitoes. Many people here do not have mosquito nets and have been sick with malaria and dengue fever, including my wife and children. I recovered from malaria IDP L7.

The remaining participants reflected on this situation. Several IDPs took refuge in their local churches and farmlands, while others were trapped in the jungle and fled to neighbouring countries’ borders. They faced numerous challenges, as the military blocked all humanitarian aid distribution from local non-governmental organisations, international organisations, and United Nations agencies. Furthermore, they are not protected from monsoon rain, making them more susceptible to flooding during the rainy season.

### Surge of infectious disease and COVID-19 cases among IDPs

The participants in this study reported that they had been living in fear since the military attacked them and had experienced an increase in infectious diseases and cases of COVID-19 in their camps. Old IDP participants in mentioned that they occasionally received health treatment from various non-governmental organisations (NGOs) before the military coup. The military, however, blocked and restricted all humanitarian supplies, including the medical workers of the NGO, from providing medical treatment, support, or protection equipment to IDP communities despite some suffering from COVID-19 symptoms. In addition, the military had arrested some philanthropic health workers who provide informal healthcare to IDPs. Therefore, the elderly population in camps are at a greater risk of contracting COVID-19 because they may have pre-existing health conditions such as paralysis, hypertension, and digestive problems.

“As an older adult living in an IDP camp, I am concerned about the welfare of elderly people. No assistance is available to older people in IDP camps, except for a monthly contribution of 15000 Kyats (9 USD) from the WFP. If patients become ill or test positive for COVID-19, 15000 kyats would not be sufficient to cover the cost of medical treatment. Since the coup, NGO workers have been unable to reach the IDP camps. This puts the elderly population in a more vulnerable condition because they are unable to receive monthly assistance owing to the failure of the banking system and roadblocks caused by the military”- IDP L5.

In particular, the participants in this study from Kachin State reported that many IDP camps had tested positive for COVID-19. They expressed their helplessness because they lacked personal protective equipment (PPE), medicine, healthcare workers, and access to healthcare facilities. This is how the camp in charge of an IDP expressed his concerns regarding a potential outbreak.

“I am afraid for the safety of everyone who tested positive for COVID-19 and the remaining camp members. We cannot perform social distancing and do not have proper handwashing facilities. There are no PPE suits, no medicine, and no vehicles to transport the patient to the hospital”- IDP L4.

With the military attack on healthcare facilities and workers after the military coup, other participants transported the patient to the nearest health centre on motorcycles and tricycles. However, they were denied hospital admission due to overcrowding and the limited number of healthcare workers during the COVID-19 outbreak in 2021. In addition, it was unaffordable for them to go to a private hospital because of higher costs and lack of financial resources. According to some participants, this forced them to rely on self-medication, resulting in numerous deaths owing to a lack of timely medical treatment.

### Lack of food supply and substantial risk of hunger

According to the participants in this study, their living conditions have become more difficult since the military coup and the subsequent political turmoil during the COVID-19 pandemic. They face food insecurity and struggle to obtain essential meals on a daily basis. As a result, they are deprived of their daily meals, and their children are already malnourished.

“We were displaced from our homes to save our lives by abandoning everything of our belongings. We do not have anything to eat in the jungle. I cannot express how hopeless I feel now.“ –IDP F10.

“We feed our children anything we can get our hands on. Little bean and rice soup now and again. Life is tough. Our children are malnourished; everything has risen following military takeovers, especially rice prices. We cannot afford to eat rice since a sack costs (40000–50000) Kyats, equivalent to (19 - 23) $ USD. Cooking oil prices have also increased. We do not have a place to borrow money. We die from starvation. ‘IDP F9

On the other hand, some IPDs have lived in camps for over ten years. Before the military coup, they could find daily basic labour to sustain their lives. They received 1,5000 kyats (9 USD) from the World Food Program (WFP) for one household, as well as other support from a variety of donors and non-governmental organisations (NGOs). As the military restricted humanitarian assistance, they experienced food insecurity. However, none of the donors, including those in the WFP, could reach them. The following are examples of how IDPs described their living conditions and vulnerability before and after the military takeover.

“Before the military coup, we could find daily labour to support ourselves. In the current climate of gunfire, heavy weaponry, and deadly pandemics, finding food is exceedingly difficult “- IDP M3.

“Due to the military coup, we have been left with nothing to eat during the COVID-19 surge. We could not obtain a monthly subsidy of 15000 Kyat (9 USD) from the World Food Programme because the military restricted all humanitarian supplies. Every day is filled with agony, as there is no source of income”- IDP F8.

### Longing peace to return home safely

The study participants reported that the military coup undermined the hope of the entire population of Myanmar. In particular, the escalating armed conflict following the military coup robbed them of their hope and dreams of returning home.

“If the military coup had not occurred, we might have been able to return to our homes and no longer be uncertain. However, we have lost everything, including our hopes and dreams. People are living in increasingly dangerous and challenging environments. Our current situation is that we do not know where to obtain food, in addition to a constant fear of being attacked **“-**IDP L5.

I wish to return to my native home. I have never been pleased by this. I am longing for the day when I will be able to return to my old house, even though it is old now”- IDP F5.

In addition, the participants in this study were concerned about returning to their homes due to military forces stationed in civilian schools, churches, and hospitals arbitrarily arresting and killing the villagers and civilians.

“The army has occupied our school and hospital buildings, so we are afraid to return home. Additionally, we learned that the military killed many civilians and arbitrarily arrested villagers. Our lives are in danger if we return home, although we go back home long.“–IDP L2.

“The military kills civilians, even in their homes, and arbitrarily arrests villagers. It is challenging for me to return home, and I am filled with anxiety beyond my word limits “-IDP F9.

## Discussion

This study explored the vulnerability and well-being of internally displaced persons (IDPs) in Myanmar following the COVID-19 pandemic and the military coup. The findings reveal that the escalation of armed clashes, mass torture, killing, and human rights violations across Myanmar has a detrimental effect on the psychological well-being of IDPs in Myanmar [31]. Moreover, the military’s indiscriminate airstrikes on civilian sites and IDP camps have resulted in many civilian fatalities, including children [32–34]. IDPs live in precarious situations because of insufficient materials for constructing camps. Furthermore, the military airstrike exacerbated their vulnerability since they sought refuge primarily in caves within the forest and under large stones. Children, women, and the elderly are particularly vulnerable because the military frequently attacks IDP camps with heavy weapons and air strikes [35, 36]. Approximately 600 military air assaults on civilians have occurred between February 2021 and January 2023 [37]. As of March 2023, over 1.9 million newly displaced persons, over half of whom were children, were affected by the conflict [6, 7, 38].

Moreover, the Myanmar military continued to use gang rape and other forms of sexual violence as weapons of war throughout the country without impunity [39, 40]. For example, a woman with a physical disability was recently raped during the military raided the village in the Sagaing Region [41]. Moreover, there have been numerous instances of gang rape and killings by the Myanmar military in the past, including killing a 37-year-old mother of four children in Rakhine State [42], killing two teachers from Kachin State in 2015 [43], and massacring Rohingya women in Rakhine State in 2017 [44]. IDP populations are, therefore, living in fear due to their concern over safety from sexual exploitation, which has detrimentally impacted their mental health and well-being.

However, the World Health Organization reported that the COVID-19 pandemic exacerbated the threat to global security and economic development, and lower public health investments undermined health and welfare worldwide [45]. However, the Myanmar military blocked lifesaving and humanitarian aid and medical workers from reaching a million internally displaced people amidst the global COVID-19 pandemic instead of providing healthcare [46]. This places individuals, especially those injured by indiscriminate airstrikes and who require medical attention, at risk of losing their lives [47–49]. Additionally, the military raided several hospitals and arrested medical personnel to provide care to the people and has been barred from access to aid and medical supplies [50–52]. As of May 2022, the Myanmar military had reported attacking over 492 health workers and destroying ambulances in the past few months. The number of arrested medical workers exceeded 564, with 126 hospitals being raided and over 36 health workers killed [53]. In this regard, lifesaving aid and healthcare workers cannot reach the IDP population in Myanmar to provide healthcare treatment. This puts the IDP population in Myanmar at a higher risk of infection with infectious diseases and contracting COVID-19 owing to a lack of access to medical and humanitarian aid. For instance, in Chin State, more than six IDPs have died because of a lack of timely healthcare treatment, including children, pregnant women, and older adults [46, 54]. Despite the lack of reliable data, the number of actual deaths seems higher nationwide. IDPs in Myanmar face two crises simultaneously: a military human rights violation and a global health catastrophe caused by COVID-19, unlike Ethiopia, whose health sector functions and humanitarian assistance, including medical supplies, are readily available [55].

As discussed above, the military coup in Myanmar worsened the challenges associated with access to healthcare treatment for IDPs. A study revealed that nearly all COVID-19 deaths in the United States were unvaccinated [56]. However, in the wake of the military coup in Myanmar, the military has consistently blocked all transportation, and the IDP cannot access COVID-19 vaccination, even though vaccine hesitancy is a global health concern in developed countries. As a result, several members of the IDP population died of COVID-19, although no reliable data were available [57]. Therefore, Myanmar IDPs are at a higher risk of becoming the epicentre of the pandemic due to a lack of vaccination and resources to protect themselves, such as personal protective equipment, soup, masks, and other prevention resources, in addition to their precarious living conditions and difficulties in accessing healthcare services.

In addition, there is a high incidence of influenza, dengue fever, and diarrhoea among children in IDP camps. In addition, many older people lack medication for high blood pressure, diabetes, and heart problems, as well as pain relievers and saline water for self-medication in case of an emergency [58]. Similarly, the participants in this study reported that they did not receive disease prevention measures, such as treatment for infectious diseases or immunisation of infants. Several participants indicated that many of their family members were infected with malaria, dengue fever, and diarrhoea due to precarious living conditions, inadequate mosquito nets, inadequate waste disposal, and lack of medication or self-medication.

In April 2020, the UN Special Rapporteur on the Human Rights of Internally Displaced Persons (IDPs) urged governments to recognise that “IDPs are the most vulnerable people and should not be forgotten by governments in their response to the pandemic’” [59]. However, Myanmar did not have a legitimate government since the military coup in 2021. The military arbitrarily arrested the president, the state councillor, and other prominent political leaders who were democratically elected to serve as government officials for the 2021–2025 term. The World Bank stated that IDPs are highly vulnerable to loss of livelihood and economic hardship compared with host communities globally [60]. The military coup in Myanmar resulted in 15.2 million individuals experiencing food insecurity in September 2022, and the World Food Programme (WFP) could only assist 2.7 million affected individuals [61]. The IDP population is particularly at risk of food insecurity due to the military blocking and restricting all international humanitarian supplies rather than providing humanitarian assistance or medical supplies to the IDP population. For instance, the military deliberately blocked the UN High Commissioner for Refugees from delivering humanitarian aid and medical supplies to Chin IDPs in Mindat Township, Chin State [62]. Thus, IDPs in Myanmar have been unable to receive humanitarian assistance, including the WFP’s existing monthly support of 15,000 kyats (9 USD). Living conditions have become difficult for them, and they do not have enough income to feed their hunger under military brutality and deadly pandemics. Their children become malnourished, and their mental health and health issues decline, significantly threatening their quality of life. In March 2021, the UNDP projected that 12 million people would live under the national poverty line by 2022 [63]. If the political turmoil in Myanmar remains unresolved, there is a significant concern that this number could escalate to 25 million or even surpass half of the country’s population by 2023. Immediate international action is crucial to mitigate the potential humanitarian crisis and address the escalating food insecurity.

## Practical policy implications

To effectively address the immediate and long-term needs of IDPs in Myanmar, encompassing protection, healthcare, food security, peacebuilding, and psychosocial support, some recommendations are proposed for international organizations, governments, non-governmental organizations, and local communities as the following:

Firstly, the findings reveal that international communities have neglected and inadequately addressed the welfare of IDPs in Myanmar. This situation will significantly impact them if humanitarian distribution remains inadequate. International humanitarian interventions are needed to address food insecurity and starvation among IDPs. Effective collaboration between international organizations, governments, and non-governmental organizations is vital to ensure consistent and sufficient food aid provision to IDP camps. This collaboration should involve establishing and utilising cross-border corridors to facilitate food aid delivery and healthcare services. Furthermore, governments and international organizations should exert pressure on the Myanmar military to refrain from attacking IDP camps and restrict humanitarian aid distribution to IDP camps.

Secondly, access to medical treatment, support, and protective equipment should be ensured in IDP communities, particularly for older individuals who are more likely to contract COVID-19. As of November 15, 2022, the global health crisis has tragically resulted in the loss of over 6.6 million lives worldwide [64]. This devastating toll also affects countries where doctors are allowed to practice, public health systems are operational, and government institutions are functioning. However, IDPs in Myanmar are more vulnerable than other global populations because of the military’s destruction of the healthcare sector and restrictions on humanitarian aid, including healthcare services. Providing prompt medical treatment and preventing unnecessary deaths among IDPs in Myanmar requires collaboration between international organizations and non-governmental organizations and establishing a mechanism to deliver healthcare services. This should include providing COVID-19 vaccines and treatment facilities to effectively combat the global health pandemic and mitigate the spread of other infectious diseases.

Thirdly, this study further highlights the adverse impact of daily challenges on women and children, particularly vulnerable to sexual abuse and violence perpetrated by the military. Therefore, it is essential for international organizations, governments, and non-governmental organizations to collaborate and advocate for the establishment of safe spaces and the implementation of measures to prevent and respond to such atrocities in line with international human rights protections. Furthermore, it is urgently necessary to establish psychosocial support programs to address IDPs’ mental health and well-being, particularly those who have experienced trauma and loss.

## Limitation and future research direction

This study has several limitations. The participants in this study were primarily from the Chin, Kachin, and the Karen States of Myanmar. Therefore, the results of this study do not represent the vulnerability of IDPs in Myanmar. Second, NGO referrals and snowball sampling methods to recruit participants and social media platforms (WhatsApp and Signal applications) to interview participants have limited the ability to connect diverse participants, given the lack of Internet and mobile signal connectivity to reach several IDP shelters. Second, despite the in-depth interviews providing a deeper understanding of the participants’ experiences, the researcher may have been influenced by translation bias in using words when transcribing audio recordings into English. Third, although the participants expressed frequent fear and anxiety about the adverse effects on their health, the findings are limited in scope for understanding the mental health coping methods among IDPs. Therefore, it is necessary to conduct more comprehensive research on the psychological condition of IDPs, their mental health, and their coping strategies using a mixed-method approach to understand their psychological state better.

## Conclusion

The IDPs in Myanmar face dual threats from military human rights violations and airstrikes, compounded by the global health COVID-19 pandemic. The increasing sexual violence against women and torture of civilians by the military following the coup has significantly increased their vulnerability, negatively impacting their mental health and overall well-being. Furthermore, the military restricted international humanitarian aid, including lifesaving and medical supplies, since the coup has exacerbated food insecurity and susceptibility to infectious diseases among IDPs. This situation has left some IDPs to lose their lives from preventable diseases since they could not access timely medical treatment services. Their precarious living conditions and unhygienic sanitation place them at substantial risk of infection with influenza, malaria, and other infectious diseases, including COVID-19. Therefore, the international community and organizations must work together to end the political crisis in Myanmar. In addition, they must provide essential humanitarian aid, including the COVID-19 vaccine, to address the unprecedented global health crisis and prevent further human catastrophes.

### Electronic supplementary material

Below is the link to the electronic supplementary material.

Supplementary Material 1: COREQ (COnsolidated criteria for REporting Qualitative research) Checklist

## Data Availability

All data generated or analysed during this study are included in this published article. The datasets generated and/or analysed during the current study are not publicly available to preserve the anonymity of study participants; however, they are available from the corresponding author upon reasonable request.
