# Supplementary material for: Vulnerability to health and well-being of internally displaced persons (IDPs) in Myanmar post-military coup and COVID-19
Source: Arch Public Health. 2023 Oct 21;81:185. doi: 10.1186/s13690-023-01204-1 (PMC10589919; doi:10.1186/s13690-023-01204-1)
Supplement: Supplementary file 1 — Supplementary Material 1: COREQ (COnsolidated criteria for REporting Qualitative research) Checklist [file 13690_2023_1204_MOESM1_ESM.pdf]

COREQ (COnsolidated criteria for REporting Qualitative research) Checklist

A checklist of items that should be included in reports of qualitative research. You must report the page number in your manuscript where you consider each of the items listed in this checklist. If you have not included this information, either revise your manuscript accordingly before submitting or note N/A.

Topic	Item No.	Guide Questions/Description	Reported on Page No.
**Domain 1: Research team and reflexivity**			
Personal characteristics			
Interviewer/facilitator	1	Which author/s conducted the interview or focus group?	Myself
Credentials	2	What were the researcher's credentials? E.g. PhD, MD	PhD Candidate in Sociology and Social Policy
Occupation	3	What was their occupation at the time of the study?	June- September 2022
Gender	4	Was the researcher male or female?	Male
Experience and training	5	What experience or training did the researcher have?	Research PhD
Relationship with participants			
Relationship established	6	Was a relationship established prior to study commencement?	No
Participant knowledge of the interviewer	7	What did the participants know about the researcher? e.g. personal goals, reasons for doing the research	No knowledge before interview. During the interview, I explained my role as a researcher and the study aim and objectives
Interviewer characteristics	8	What characteristics were reported about the inter viewer/facilitator? e.g. Bias, assumptions, reasons and interests in the research topic	Interest in the research topic. They expressed mental stress relief after the interview.
**Domain 2: Study design**			
Theoretical framework			
Methodological orientation and Theory	9	What methodological orientation was stated to underpin the study? e.g. grounded theory, discourse analysis, ethnography, phenomenology, content analysis	No specific methodological orientation.
Participant selection			
Sampling	10	How were participants selected? e.g. purposive, convenience, consecutive, snowball	Purposive, snowball and NGO's referrals
Method of approach	11	How were participants approached? e.g. face-to-face, telephone, mail, email	Online (Zoom or WhatsApp)
Sample size	12	How many participants were in the study?	7 IDP leaders and 10 IDP members
Non-participation	13	How many people refused to participate or dropped out? Reasons?	I do not have the record. I only contacted to participants who give consent to participate voluntary to the NGO or IDPs camp leaders.

Setting			
Setting of data collection	14	Where was the data collected? e.g. home, clinic, workplace	IDPs camp from Kachin, Chin and Karen State in Myanmar
Presence of nonparticipants	15	Was anyone else present besides the participants and researchers?	No
Description of sample	16	What are the important characteristics of the sample? e.g. demographic data, date	Participants are IDP camp leaders and members themselves instead of NGOs who might not have the experience and suffer like the IDP.
Data collection			
Interview guide	17	Were questions, prompts, guides provided by the authors? Was it pilot tested?	Yes, this is a kind of a pilot test to understand the plights and mental health situation of IDPs in Myanmar.
Repeat interviews	18	Were repeat inter views carried out? If yes, how many?	No
Audio/visual recording	19	Did the research use audio or visual recording to collect the data?	Both
Field notes	20	Were field notes made during and/or after the inter view or focus group?	Some note was taken during the interview.
Duration	21	What was the duration of the inter views or focus group?	25-35 minutes in average
Data saturation	22	Was data saturation discussed?	Yes
Transcripts returned	23	Were transcripts returned to participants for comment and/or	No. They cannot understand English
**Topic**	**Item No.**	**Guide Questions/Description**	**Reported on Page No.**
		correction?	
**Domain 3: analysis and findings**			
Data analysis			
Number of data coders	24	How many data coders coded the data?	Myself
Description of the coding tree	25	Did authors provide a description of the coding tree?	Yes
Derivation of themes	26	Were themes identified in advance or derived from the data?	Mainly derived from data
Software	27	What software, if applicable, was used to manage the data?	No, the data were analysed manually
Participant checking	28	Did participants provide feedback on the findings?	No, this condition was not possible due to internet signal problem and they do not understand English.
Reporting			
Quotations presented	29	Were participant quotations presented to illustrate the themes/findings?	Yes, L = Leader

		Was each quotation identified? e.g. participant number	
Data and findings consistent	30	Was there consistency between the data presented and the findings?	Yes
Clarity of major themes	31	Were major themes clearly presented in the findings?	Yes
Clarity of minor themes	32	Is there a description of diverse cases or discussion of minor themes?	No specific diverse cases are found.

Developed from: Tong A, Sainsbury P, Craig J. Consolidated criteria for reporting qualitative research (COREQ): a 32-item checklist for interviews and focus groups. *International Journal for Quality in Health Care*. 2007. Volume 19, Number 6: pp. 349 – 357
